# Correction: Mathematical Modeling of Influenza A Virus Dynamics within Swine Farms and the Effects of Vaccination

**DOI:** 10.1371/journal.pone.0111832

**Published:** 2014-11-20

**Authors:** The PLOS ONE Staff

## Notice of Republication

This article was republished on October 14, 2014, to correct typesetting errors involving missing equations throughout the text and tables. The publisher apologizes for these errors. Please download this article again to view the corrected version. The originally published, uncorrected article and the republished, corrected article are provided here for reference.

## Supporting Information

File S1 Originally published, uncorrected article. 27 August, 2014
(PDF)

File S2 In progress, partially corrected article. 14 October, 2014
(PDF)

File S3 Republished, corrected article. 9 March, 2015
(PDF)
