# Supplementary material for: Correction: Mathematical Modeling of Influenza A Virus Dynamics within Swine Farms and the Effects of Vaccination
Source: PLoS One. 2014 Nov 20;9(11):e111832. doi: 10.1371/journal.pone.0111832 (PMC4239039; doi:10.1371/journal.pone.0111832)
Supplement: File S1 — Originally published, uncorrected article. 27 August, 2014 (PDF) [file pone.0111832.s001.pdf]

Mathematical Modeling of Influenza A Virus Dynamics within Swine Farms and the Effects of Vaccination

Jennifer J. H. Reynolds*, Montserrat Torremorell, Meggan E. Craft

Department of Veterinary Population Medicine, University of Minnesota, St Paul, Minnesota, United States of America

Abstract

Influenza A virus infections are widespread in swine herds across the world. Influenza negatively affects swine health and production, and represents a significant threat to public health due to the risk of zoonotic infections. Swine herds can act as reservoirs for potentially pandemic influenza strains. In this study, we develop mathematical models based on experimental data, representing typical breeding and wean-to-finish swine farms. These models are used to explore and describe the dynamics of influenza infection at the farm level, which are at present not well understood. In addition, we use the models to assess the effectiveness of vaccination strategies currently employed by swine producers, testing both homologous and heterologous vaccines. An important finding is that following an influenza outbreak in a breeding herd, our model predicts a persistently high level of infectious piglets. Sensitivity analysis indicates that this finding is robust to changes in both transmission rates and farm size. Vaccination does not eliminate influenza throughout the breeding farm population. In the wean-to-finish herd, influenza infection may persist in the population only if recovered individuals become susceptible to infection again. A homologous vaccine administered to the entire wean-to-finish population after the loss of maternal antibodies eliminates influenza, but a vaccine that only induces partial protection (heterologous vaccine) has little effect on influenza infection levels. Our results have important implications for the control of influenza in swine herds, which is crucial in order to reduce both losses for swine producers and the risk to public health.

Citation: Reynolds JJH, Torremorell M, Craft ME (2014) Mathematical Modeling of Influenza A Virus Dynamics within Swine Farms and the Effects of Vaccination. PLoS ONE 9(8): e106177. doi:10.1371/journal.pone.0106177

Editor: Alain Barrat, Centre de Physique Théorique, France

Received April 21, 2014; **Accepted** July 30, 2014; **Published** August 27, 2014

Copyright: © 2014 Reynolds et al. This is an open-access article distributed under the terms of the Creative Commons Attribution License, which permits unrestricted use, distribution, and reproduction in any medium, provided the original author and source are credited.

Data Availability: The authors confirm that all data underlying the findings are fully available without restriction. All relevant data can be accessed from articles cited within the paper.

Funding: This material is based upon work supported by the University of Minnesota's Institute on the Environment and the Cooperative State Research Service, U.S. Department of Agriculture, under Project No. MINV 62-042. The funders had no role in study design, data collection and analysis, decision to publish, or preparation of the manuscript.

Competing Interests: The authors have declared that no competing interests exist.

* Email: jblack@umn.edu

Introduction

Influenza infections are some of the most costly and deadly zoonoses because of the virus's pathogenicity and ability to rapidly spread and evolve. Influenza A virus is notable for its complex ecology involving multiple avian and mammalian hosts. Specifically, all human influenzas in recent history have involved viruses of avian or swine origin [1]. Pigs pose a particular threat as "mixing vessels" for generating new viral strains through reassortment of human, swine, and avian viruses [2]; swine farms can act as reservoirs for influenza strains with pandemic potential [3]. Influenza A virus is ubiquitous in global pig populations [4,5], causing acute respiratory disease in pigs [6] and negatively affecting swine production [7]. It is therefore timely and critical to understand influenza dynamics and the mechanisms of influenza persistence in swine farms, not only to reduce losses for producers, but also to reduce the risk of emerging zoonotic strains. Understanding the on-farm epidemiological dynamics of influenza can result in improved methods of control and the prevention of outbreaks.

Influenza A virus is highly contagious, with transmission between pigs occurring via several different routes [5]. Transmission routes include direct contact with infected pigs [6,8], aerosols [9], and exposure to contaminated fomites [10]. Influenza transmission depends on multiple factors, including swine age, immunity, vaccination status and the presence of maternal antibodies. Vaccination is commonly used as a control measure for influenza in swine farms [11]. Approximately 70% of large producers in the U.S. reported that they vaccinated breeding females for influenza in 2006 [12], and approximately 20% reported that they vaccinated weaned pigs [12]. Vaccination has been shown to reduce influenza A virus transmission in pigs in experimental settings [13,14], but the effects of vaccination at the farm level remain unclear. Maternally-derived immunity, passed from immune sows to their offspring by means of colostrum, can also reduce transmission of influenza virus [13,15]. This maternal immunity in piglets wanes over time [16]. Due to these multiple factors, influenza A virus transmission is complex, and there is an overall lack of understanding of transmission dynamics at the population level. In addition, the thorough empirical assessment of infection levels on a herd level through time is impractical and costly, and consequently empirical data on influenza dynamics at the farm-scale is lacking.

Mathematical modeling is a practical and useful tool for understanding disease dynamics, explaining observed patterns based on mechanistic hypotheses, and testing possible control measures [17]. Although several mathematical modeling studies have focused on infectious diseases in swine (e.g. Nipah virus [18],

pseudorabies [19,20], porcine reproductive and respiratory syndrome virus [21], and Salmonella [22,23]), there is a lack of modeling studies on influenza in swine herds. Indeed, a recent review of the literature carried out by Dorjee et al. [24] found no manuscripts reporting the modeling of influenza in swine, despite the importance of influenza and its global ubiquity in swine populations.

In order to provide insights into influenza dynamics at the herd level, we build mathematical models for two types of swine farm: a breeding farm and a wean-to-finish facility. These models are based on, and parameterized by, recent experimental data on influenza. In particular, new studies on influenza transmission in experimental settings have provided values for transmission rates [10,13,14], often a difficult parameter to estimate [25]. For the breeding farm, we include the typical components of a standard U.S. commercial farm, including the spatial separation of swine into different subpopulations. These spatial subpopulations are represented by a metapopulation model, which is further structured by swine class; a class is defined as a weekly stage in the swine production process, where each week swine progress to the next appropriate class. Each swine class in all spatial locations (e.g. gilt development units, farrowing rooms and gestation areas) is thus explicitly modeled. This model framework allows for incorporation of heterogeneities in the influenza transmission rate across the population as seen in experimental studies, specifically differences in transmission imposed by the farm spatial structure and also in respect to the swine class. For the wean-to-finish farm, we also use a class-structured model; however, spatial separation of the animals is not required since all animals are housed in the same building.

For influenza in pigs, once a susceptible animal becomes infected, there is an exposed or latent period of approximately 2 days [24], when the animal is infected but not yet infectious. This is followed by an infectious period lasting an average of 5 days [3,14,24,26], after which the animal recovers. The models presented here therefore comprise series of *SEIR* (susceptible, exposed, infectious, recovered) ordinary differential equations, made specific to the swine – influenza system. Such deterministic differential equation models have been used to gain insights into the dynamics of other swine diseases within farms [18–20,22]. Modified versions of the *SEIR* approach have also been used to model influenza in human populations (e.g. [27–29]). Our models are designed to capture the infection dynamics for the large population numbers of swine on the farms, and are intended to investigate general trends (for instance, infection peaks and endemicity) as opposed to precise quantitative predictions.

In this study, we develop new models designed to represent the essential features of swine farms and the epidemiology of influenza. Our goal is to predict typical infection dynamics at the population scale, offering important clarification of this complex system. Based on a recent experimental study that estimated that piglets have the highest infection levels of influenza in one swine herd [30], we hypothesize that piglets might be the subpopulation of swine with the highest infection levels on swine farms in general. In particular, we aim to (a) describe typical infection dynamics, as these are as yet unknown for influenza in swine farms; (b) explore the possibility of reinfection and the maintenance of influenza virus in swine populations through time; (c) identify the class(es) of pigs most likely to have high infection levels; and (d) evaluate the effectiveness of common vaccination strategies in reducing the level of influenza infection.

Methods

Construction of Breeding Farm Model

The model is structured to represent the management of a standard commercial swine breeding farm. We have incorporated typical swine demographic rates and structural features of the farm, which are illustrated in Figure 1. The model farm houses gilts, sows and piglets and includes an on-site gilt development unit (GDU) as well as separate buildings for the breeding/gestation and farrowing areas. The model captures both the physical structure of the farm and the movement of swine through the farm. The movement of swine through the farm is not continuous because animals are moved together in groups at regular intervals. Due to the spatial partitioning of the farm, we use a *metapopulation model*. This type of model enables the incorporation of spatial structure in a population, and thus the incorporation of differences in transmission rates (for instance, transmission within a spatial unit can be different to transmission between units) [31,32]. Furthermore, the swine farm population comprises animals of different age or reproductive status; these animals can be housed together in the same room, for instance, sows with piglets in the farrowing rooms, and weaned sows with pregnant sows in the breeding/gestation area. To represent this organizational structure, and because transmission rates can vary between different types of animal, we group individuals into classes, and model each class explicitly. Specifically, we define a *class* to be a weekly stage in the swine production process.

In line with experimental evidence, our model includes both direct transmission between pigs in the same room [14], and indirect transmission between pigs in separate rooms [10]. Infection can also spread in the model by the physical movement of swine through the farm.

Our breeding farm model couples a continuous-time epidemiological model and a discrete-time (discontinuous) population model. For the epidemiological model, we define separate series of *SEIR* differential equations for each class of swine. Firstly, for sows and gilts:

$$\frac{dS_i}{dt} = -\beta_{\mathrm{d}} I_{\mathrm{d}_i} S_i - \beta_{\mathrm{ind}} I_{\mathrm{ind}_i} S_i - \mu S_i \qquad (1)$$

$$\frac{dE_i}{dt} = \beta_{\mathrm{d}} I_{\mathrm{d}_i} S_i + \beta_{\mathrm{ind}} I_{\mathrm{ind}_i} S_i - (\mu + \sigma) E_i \qquad (2)$$

$$\frac{dI_i}{dt} = \sigma E_i - (\mu + \gamma) I_i \qquad (3)$$

$$\frac{dR_i}{dt} = \gamma I_i - \mu R_i \qquad (4)$$

Here S_i represents the number of susceptible animals in class i, E_i the number of exposed animals (i.e. infected but not yet infectious), I_i the number of infectious animals, and the number of animals that have recovered from infection (and are therefore immune). All animals are characterized as belonging to one of these disease states (S, E, I, R). The class of pigs corresponding to each index i is given in Table 1. The variable I_{d_i} is defined as the total number of infectious animals in the same room as class (including infectious piglets); the variable I_{ind_i} is defined as the total number of infectious animals in all other rooms on the farm (including infectious piglets). For instance, for gilt class 1, the variable I_{d_1} would be the total number of infectious gilts in the

GDU (i.e. those in classes 1 to 10), and $I_{\text{ind}\,1}$ would be the total number of infectious animals in all locations of the farm excluding the GDU. Parameter β_{d} is the direct transmission rate, and β_{ind} the indirect transmission rate. We assume density-dependent transmission [33], because influenza transmission likely increases with swine population density. The parameters involved in these equations are defined in Table 2. This model does not include additional mortality due to influenza infection, as this rate is generally very low in practice (<1%) [11,34].

Secondly, for piglets, we separate the population into two groups: those that have maternally-derived immunity and those that do not. We assume that the decay of maternal immunity starts at 3 weeks of age [16]. As piglets in the breeding farm are younger than this threshold, we keep the transmission rate for piglets with maternal immunity the same through time in this model. (We include the waning immunity demonstrated for older animals in our wean-to-finish model.) The equations for piglets without maternal immunity are:

$$\frac{dS_j^{\text{p}}}{dt}=b_j(S_{i=27}+E_{i=27}+I_{i=27})-\beta_{\text{d}}^{\text{p}}I_{\text{d}_j}S_j^{\text{p}}-\beta_{\text{ind}}^{\text{p}}I_{\text{ind}_j}S_j^{\text{p}}-\mu^{\text{p}}S_j^{\text{p}} \quad (5)$$

$$\frac{dE_j^{\text{p}}}{dt}=\beta_{\text{d}}^{\text{p}}I_{\text{d}_j}S_j^{\text{p}}-\beta_{\text{ind}}^{\text{p}}I_{\text{ind}_j}S_j^{\text{p}}-(\mu^{\text{p}}+\sigma)E_j^{P} \quad (6)$$

$$\frac{dI_j^{\text{p}}}{dt}=\sigma E_j^{\text{p}}-(\mu^{\text{p}}+\gamma)I_j^{\text{p}} \quad (7)$$

$$\frac{dR_j^{\text{p}}}{dt}=\gamma I_j^{\text{p}}-\mu^{\text{p}}R_j^{\text{p}} \quad (8)$$

and those for piglets with maternal immunity are:

$$\frac{dS_j^{\text{pm}}}{dt}=b_j(R_{i=27})-\beta_{\text{d}}^{\text{pm}}I_{\text{d}_j}S_j^{\text{pm}}-\beta_{\text{ind}}^{\text{pm}}I_{\text{ind}_j}S_j^{\text{pm}}-\mu^{\text{p}}S_j^{\text{pm}} \quad (9)$$

$$\frac{dE_j^{\text{pm}}}{dt}=\beta_{\text{d}}^{\text{pm}}I_{\text{d}_j}S_j^{\text{pm}}+\beta_{\text{ind}}^{\text{pm}}I_{\text{ind}_j}S_j^{\text{pm}}-(\mu^{\text{p}}+\sigma)E_j^{\text{pm}} \quad (10)$$

$$\frac{dI_j^{\text{pm}}}{dt}=\sigma E_j^{\text{pm}}-(\mu^{\text{p}}+\gamma)I_j^{\text{pm}} \quad (11)$$

$$\frac{dR_j^{\text{pm}}}{dt}=\gamma I_j^{\text{pm}}-\mu^{\text{p}}R_j^{\text{pm}} \quad (12)$$

for $j=$ 1, 2, 3, 4, where each indexed class of piglets is located in a different room in the farrowing area. Superscripts p and pm are used to identify piglets without maternal immunity and piglets with maternal immunity, respectively. Here, and R_{27} are the numbers of susceptible, exposed, infectious and recovered animals in class $i=$ 27, i.e. sows due to farrow. Parameter b_j is the birth rate, defined so that sows give birth to an average number of 12 piglets

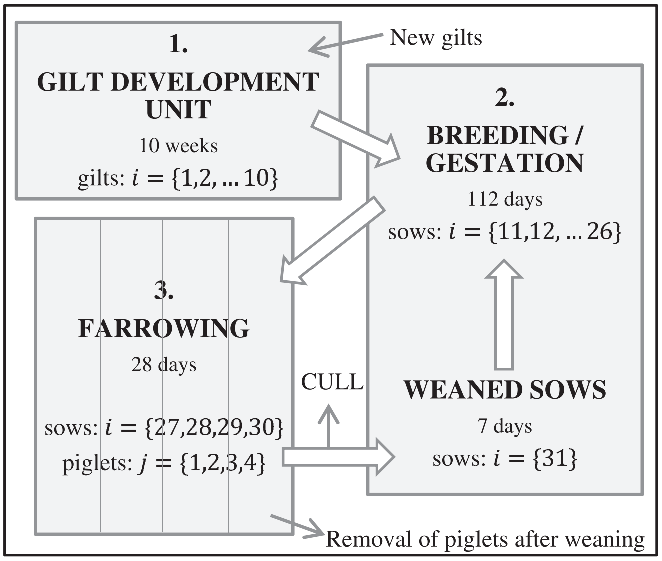

Figure 1. Schematic of a standard commercial swine breeding farm showing the demographic and spatial structure assumed in our mathematical model. This farm houses *gilts* (female pigs that have not yet been mated), *sows* (female pigs) and *piglets* (young pigs). There are three separate buildings (indicated by the shaded boxes), and the farrowing building is subdivided into four rooms. *Farrowing* means the production of a litter of piglets, and *weaning* is the separation of a sow and her piglets. New gilts enter the gilt development unit (building 1) at a replacement sow rate of 50% year^{-1}. From here, animals are moved to building 2 and inseminated. Typically, swine farmers rely primarily on artificial insemination for breeding and house only a small number of boars, thus we have excluded boars from the model. After 112 days, pregnant sows are moved to building 3, where 2–7 days later they give birth to an average of 12 piglets per sow. Sows remain in building 3 for 28 days, and then are moved back to building 2. After one week, insemination takes place again, and this cycle continues. Weaning occurs twice a week. After weaning, piglets are removed from the breeding farm. The overall death/removal rate for sows is 50% year^{-1}, with 80% of this occurring after weaning at the cull of unproductive sows. The natural death rate for piglets is 10% from birth to weaning. Class indices *i* (gilts and sows) and *j* (piglets) (see Table 1) are indicated.
doi:10.1371/journal.pone.0106177.g001

Table 1. The class of pigs (type and farm location) corresponding to each index value for the breeding farm model.

Class index	Animal type	Farm location (see Figure 1)
$i \in \{1,2,\ldots 10\}$	Gilts	1. Gilt Development Unit
$i \in \{11,12,\ldots 26\}$	Pregnant sows	2. Breeding/Gestation Area
$i \in \{27,28,29,30\}$	Farrowing and lactating sows	3. Farrowing Area
$i \in \{31\}$	Weaned sows	2. Breeding/Gestation Area
$j \in \{1,2,3,4\}$	Piglets	3. Farrowing Area

For example, $i = 12$ corresponds to sows in their second week of pregnancy, housed in the breeding/gestation area.
doi:10.1371/journal.pone.0106177.t001

between days 2 and 7 each week, into piglet class . Thus $b_j = 12/5$ for and $b_j = 0$ otherwise. This yields an average number of 30 piglets born per sow per year. See Table 2 for descriptions of all model parameters.

Other population processes occurring on the breeding farm are modeled discretely. We assume that physical movement of pigs through the farm occurs weekly. The continuous breeding farm model equations ((1)–(12)) run for one week, and then initial conditions are reset in agreement with the progression of animals through the farm according to Figure 1, i.e. swine progress to the next weekly class. In addition, at the start of every week, new gilts enter the GDU into class , and sows are culled after weaning. For piglet class $j = 1$, at the start of each week, initial conditions are set to $\left(S_1^{p}, E_1^{p}, I_1^{p}, R_1^{p}\right) = \left(S_1^{pm}, E_1^{pm}, I_1^{pm}, R_1^{pm}\right) = (0,0,0,0)$. For the oldest piglet class (class $j = 4$), weaning occurs twice weekly; half of the piglets are removed midweek, and the remainder removed at the start of each new week.

For this study, we assume there are approximately 2500 sows and gilts on the breeding farm (and test the sensitivity of our findings to this number; see 'Varying Farm Size' section), out of which approximately 320 are gilts. This yields approximately 1100 weaned piglets each week and a total of approximately 3300 to 4100 piglets on the farm. The model was coded and run in MATLAB (2012; www.mathworks.com), using differential equation solver *ode45*. If the total number of exposed and infectious pigs falls below one, the infection was considered to have gone extinct. For the purposes of this paper, we assume that introduction of the virus into the breeding farm is by the entry of one infected gilt into the GDU, and that all other animals are susceptible. We assume there is a single influenza strain in the farm; therefore we limit our timescales (to 40 days) to capture the infection dynamics of a single strain.

The use of this 'class-structured' metapopulation model allows us to obtain a complete picture of influenza transmission through the swine breeding farm. The separation of the total swine population into multiple classes also allows us to model the implementation of vaccination strategies involving vaccination of a specific group of swine (see 'Vaccination' subsection). Our model captures the essential dynamical features of the swine – influenza system, i.e. influenza transmission is continuous while the movement of swine is discontinuous.

Model with variability in transmission. In order to evaluate the robustness of our findings to variation in influenza transmission rates, we examined the effects of changing the direct and indirect transmission rates (those marked with a * in Table 2) within their 95% confidence interval ranges. For each transmission rate, we randomly sampled from a uniform distribution spanning its 95% confidence interval. This sampling was performed for all transmission rates simultaneously for each run of the model and we ran this sensitivity model 15,000 times.

Vaccination. We used our swine breeding farm model to test the effectiveness of the two most common vaccination protocols used at swine breeding farms. The first is *mass vaccination*, where all sows and gilts are vaccinated at one time, or within a very short time period. The second is *pre-farrow vaccination*, where all pregnant sows are vaccinated 5 and 3 weeks prior to farrow, to target the transfer of maternal immunity to piglets. The pre-farrow vaccination is therefore an ongoing procedure, as opposed to the mass vaccination. For each protocol, vaccines can be either *homologous* or *heterologous*. Homologous here refers to an autogenous vaccine prepared with the isolate recovered from the specific population in which it will be used. In contrast, heterologous vaccines refer to vaccines prepared with isolates distinct from the specific strain in the population. Isolates in heterologous vaccines may induce variable degrees of cross-protection against circulating strains.

The transmission parameter (β) values used to model these vaccination strategies originate from experimental studies where influenza transmission is measured from an infected pig to vaccinated pigs [14] or to piglets born from vaccinated sows [13]. Transmission values were calculated for both homologous and heterologous vaccines, and are given in Table 2. Our model (equations (1)–(12)) is adapted for each vaccination type, by replacing the appropriate transmission (β) values for the appropriate classes of swine.

We model two different scenarios with regards to the timing of vaccination, both of which could occur in the field. Firstly, we test the effects of vaccination occurring **prior** to the introduction of the virus. We make the assumption that influenza infection occurs prior to the decay of vaccine efficacy. Secondly, we model the effects of vaccination **after** an outbreak, i.e. when influenza is endemic and circulating and some of the population is immune (after the infection peak, when the system is at equilibrium).

Sensitivity analysis. To analyze the sensitivity of our results to variation in transmission rates of vaccinated animals, we ran the model with the implementation of vaccination for direct transmission parameters (those marked ** in Table 2) across the range of their 95% confidence intervals.

Construction of Wean-To-Finish Farm Model

The wean-to-finish farm is populated with *weaned pigs* (i.e. piglets that have been moved from a breeding farm after weaning) following all-in/all-out procedures. These animals are kept at the wean-to-finish facility to grow for 6 months until they are sold to slaughter. In contrast to the breeding farm, in the wean-to-finish farm all animals are considered well-mixed due to the lack of spatial separation; all animals are housed together in large pens in one building. Direct transmission of influenza can occur between all pigs in the wean-to-finish farm. Here we have assumed that the total number of animals in this farm is 2000, and later test the

Table 2. Parameters involved in the swine breeding farm model, with definitions, values and the sources of the values.

Model parameter	Meaning	Value	Experimental source of value
**DISEASE PARAMETERS**			
β_{d}	Direct transmission rate for sows and gilts	0.285 day^{-1} *(95% confidence interval: 0.091–0.9)	Experimental data from Romagosa et al. [14] (mean of transmission values from non-vaccinated treatment group)
β_{ind}	Indirect transmission rate for sows and gilts	$(=\beta_{\mathrm{d}}/178)$0.0016 day^{-1} *	Calculated from experimental data from Allerson et al. [10]
$\beta_{\mathrm{d}}^{\mathrm{p}}$	Direct transmission rate for piglets	0.218 day^{-1} *(95% confidence interval: 0.147–0.310)	Experimental data from Allerson et al. [13] (transmission rate for non-vaccinated treatment group)
$\beta_{\mathrm{ind}}^{\mathrm{p}}$	Indirect transmission rate for piglets	0.001 day^{-1} *	Extrapolation from experimental data from Allerson et al. [10]; we assume
$\beta_{\mathrm{d}}^{\mathrm{pm}}$	Direct transmission rate for piglets with maternal immunity	0.014 day^{-1} *(95% confidence interval: 0.001–0.061)	Experimental data from Allerson et al. [13]
$\beta_{\mathrm{ind}}^{\mathrm{pm}}$	Indirect transmission rate for piglets with maternal immunity	0.00008 day^{-1} *	Extrapolation from experimental data from Allerson et al. [10]
σ	Reciprocal of average duration of latent/exposed period	1/2 day^{-1}	Survey of swine influenza literature [24]
γ	Reciprocal of average duration of infectious period (or recovery rate)	1/5 day^{-1}	Survey of swine influenza literature [3,14,24,26]
**VACCINATION PARAMETERS**			
	Direct transmission rate for pigs vaccinated with heterologous vaccine	0.0275 day^{-1} **(95% confidence interval: 0.001–0.115)	Experimental data from Romagosa et al. [14] (mean of transmission values from heterologously vaccinated treatment group)
	Direct transmission rate for pigs vaccinated with homologous vaccine	0 day^{-1} **(95% confidence interval: 0–0.052)	Experimental data from Romagosa et al. [14] (no transmission occurred to pigs in homologously vaccinated treatment group)
	Direct transmission rate for piglets when mother vaccinated with heterologous vaccine	0.174 day^{-1} **(95% confidence interval: 0.118–0.246)	Experimental data from Allerson et al. [13] (transmission rate from heterologously vaccinated treatment group)
	Direct transmission rate for piglets when mother vaccinated with homologous vaccine	0.014 day^{-1} **(95% confidence interval: 0.001–0.061)	Experimental data from Allerson et al. [13] (transmission rate for homologously vaccinated treatment group)
Indirect transmission rates for each vaccine type are extrapolated from experimental data from Allerson et al. [10].			
**SWINE FARM PRODUCTION PARAMETERS**			
b	Birth rate	12 live births per litter per sow (between days 2 and 7 each week)	From expert knowledge of swine farm and PigCHAMP website (www.pigchamp.com/LinkClick.aspx?fileticket=NMdM5F73gKE%3d&tabid=115)
μ	Natural death rate for sows and gilts (i.e. death rate not including culled animals)	0.0004 day^{-1}	When combined with the cull, this value gives a total death rate of 50% per year (from expert knowledge of swine farm and PigCHAMP website)
μ^{p}	Natural death rate for piglets	0.005 day^{-1}	Corresponds to a death rate of 10% from birth to weaning (from expert knowledge of swine farm and PigCHAMP website)

The point/exact transmission values are used for the main simulations presented in this paper. "*" indicate transmission values that are varied across the range of their 95% confidence intervals for the model with variability in transmission. "**" similarly indicate the vaccination parameters that are varied for a sensitivity analysis.
doi:10.1371/journal.pone.0106177.t002

sensitivity of our findings to this number. The natural death rate for these pigs is 5% per 6 months.

For the purposes of our model, weaned pigs populate the wean-to-finish farm over a 3-week period. We make the assumption that 3 equally-sized batches of pigs enter the farm, one per week during this period. We use three series of $SEIR$ differential equations, one for each of these batches of weaned pigs:

$$\frac{dS_k}{dt} = \beta(I_1 + I_2 + I_3)S_k - \mu^{\mathrm{w}} S_k \quad (13)$$

$$\frac{dE_k}{dt} = \beta(I_1 + I_2 + I_3)S_k - (\mu^{\mathrm{w}} + \sigma)E_k \quad (14)$$

$$\frac{dI_k}{dt} = \sigma E_k - (\mu^{\mathrm{w}} + \gamma)I_k \quad (15)$$

$$\frac{dR_k}{dt} = \gamma I_k - \mu^{\mathrm{w}} R_k \quad (16)$$

for $k = 1, 2, 3$. Pigs are at weaning age (21 days) when they enter the wean-to-finish farm. When the farm is fully populated, each of the three batches of pigs will be of a different age. Because maternal immunity begins to wane at 21 days [16], the three batches of pigs will have different levels of maternal immunity. We therefore model the three classes (indexed by k) separately. To represent the waning of maternal immunity, we use a time-dependent transmission rate, $\beta = \beta_{\mathrm{m}}(T)$, for pigs that have maternal immunity (see Table 3). Here, time is defined as the time since the pig entered the wean-to-finish farm (i.e. pig –21 days) and the rate is quantified by experimental evidence from Markowska-Daniel et al. [16]. The parameters featured in this wean-to-finish model are explained in Table 3.

As with the breeding farm model, these model equations were coded and run in MATLAB. We assume that one infectious weaned pig enters the wean-to-finish farm with the third group (i.e. once the farm is fully populated), and that the rest of the farm population is susceptible. We chose to introduce one pig to represent the minimum infection prevalence at weaning. The time that the infectious pig enters the farm is set as time $t = 0$.

Reinfection. We modeled two scenarios involving the possibility of pig reinfection with influenza. Firstly, there is evidence that pigs with maternal antibodies that get infected with influenza do not develop full immunity once recovered, and are thus able to be reinfected [35]. We model this scenario by allowing animals that are infected early in life (specifically, in the two weeks after the introduction of influenza virus), to be able to reenter the susceptible pool once recovered.

Secondly, the influenza virus strain could potentially change via mutation over time, so that recovered individuals become susceptible to infection once more. Or, recovered individuals may reenter the susceptible class due to the decay of antibodies. We test a modification of model equations (13)–(16), involving the introduction of a new parameter, ω , the average rate at which recovered animals become susceptible. Specifically, a $+\ \omega R_k$ term is added to equation 13, and a $-\ \omega R_k$ term added to equation 16.

Vaccination. Maternal immunity can interfere with vaccination, and block its efficacy [36], thus vaccination in wean-to-finish pigs typically takes place after the decay of maternal antibodies [12]. We implemented vaccination in our wean-to-finish model by replacing the transmission rates (β) with the transmission rates for vaccinated pigs (or ; shown in Table 3), 10 weeks after the farm becomes fully populated. At this time, all pigs will be at least 13 weeks old, and maternal antibodies will have decayed [16]. We tested both heterologous and homologous vaccines.

Varying Farm Size

The sizes of breeding and wean-to-finish farms can vary, and this may affect disease dynamics [20]. We therefore use our models to predict influenza dynamics across a wide range of different farm sizes. For the breeding farm, we experiment with a range of sizes from 250 to 5000 sows and gilts. For the wean-to-finish farm, we use group sizes ranging from 250 to 3500 weaned pigs. When the farm size is varied, the number of classes is assumed to stay the same, and the number of animals in each class scaled accordingly. To determine if our influenza dynamics results were sensitive to commercial farm size, two measures of influenza infection dynamics were calculated: maximum proportion of infectious piglets at equilibrium on a breeding farm, and proportion of infectious pigs at the infection peak on a wean-to-finish farm. Equilibrium here refers to the number of pigs in each of the S, E, I, R infection states after the infection peak, where dynamics remain steady through time.

Results

Influenza Dynamics in the Breeding Farm

Our model predicts that in a naïve population, influenza spreads rapidly throughout the breeding farm following the introduction of an infectious gilt (Figure 2). There is an initial peak in the number of infectious animals, and a rapid decline in the number of susceptible ones. At the infection peak for sows and gilts, approximately half of the animals are infectious. The majority of the infectious animals are sows, with only 0.1% of them being gilts. After the initial peak, the population settles to an equilibrium (Figure 2a); the post-epidemic equilibrium population comprises mainly recovered animals, with a low persistent level of infectious animals. Specifically, there are approximately 20–30 infectious animals (~1% of the total population of sows and gilts), out of which the majority (15–27, or ~70–90%) are gilts. In contrast, for piglets, the equilibrium behavior is cyclic, with a relatively high level of infectious animals (between ~660 and 960 piglets, or 18–27% of the total piglet population) (Figure 2b). This cyclicity is due to the interaction between the dynamical process of influenza infection and the regular birth of new susceptible animals.

Model with variability in transmission

When variation in the direct and indirect transmission rates was incorporated, similar qualitative patterns in the number of infectious animals were observed (Figure 3). Although there is little variation in the number of infectious sows and gilts at equilibrium, there is some variation in the timing of the initial outbreak (Figure 3a). For piglets, there is comparatively less variation in the timing of the initial outbreak and large variation in the number of infectious piglets after the infection peak (Figure 3b). Despite the large variation, influenza infection is still maintained in the piglet population.

Testing vaccination strategies

Figure 4 illustrates the results when vaccination occurs prior to the introduction of influenza. For sows and gilts (Figure 4a), in both mass and pre-farrow vaccination strategies with heterologous vaccines, there is a slight delay in the outbreak and a slight reduction in the number of infectious animals at the infection peak. Mass vaccination with a homologous vaccine eliminates influenza in sows and gilts, as would be expected. Pre-farrow vaccination with a homologous vaccine reduces the number of infectious animals at the peak by approximately a third, and delays the outbreak. However, it causes the tail of the outbreak to include more infectious individuals, resulting in an increased number of infectious sows and gilts after the infection peak. For piglets (Figure 4b), the homologous pre-farrow vaccination delays the initial outbreak. No vaccination strategy significantly reduces the number of infectious piglets at equilibrium.

When vaccination is implemented when the farm is already infected, i.e. when influenza is endemic and circulating, the pre-farrow homologous vaccine no longer causes an increase in the number of infectious sows and gilts (Figure S1a; see Supporting Information) and the number of infectious piglets is still not measurably reduced by vaccination (Figure S1b).

We tested how robust these vaccination results are to uncertainties in transmission estimates, and found that varying direct transmission rates did not change the qualitative trends of the number of infectious animals in the vaccination predictions, except in the homologous mass vaccination case, where influenza was no longer eliminated in sows and gilts for direct transmission rate values in the upper part of the 95% confidence interval. All

Table 3. Parameters involved in the wean-to-finish farm model, with definitions, values and the sources of the values.

Model parameter	Meaning	Value	Experimental source of value
**DISEASE PARAMETERS**			
	Transmission rate for pigs with maternal immunity from immune mothers (time-dependent due to the decay of maternal antibodies)	$-0.273e^{-0.06T}+0.287$ day^{-1}	Extrapolation from data from Markowska-Daniel et al. [16] (fitting to the control data from H1N1 strain decay of antibodies); T = time elapsed since pig entered farm
β	Transmission rate for pigs without maternal immunity	0.285 day^{-1}	Experimental data from Romagosa et al. [14] (mean of transmission values from non-vaccinated treatment group)
σ	Reciprocal of average duration of latent/exposed period	1/2 day^{-1}	Survey of swine influenza literature [24]
γ	Reciprocal of average duration of infectious period (or recovery rate)	1/5 day^{-1}	Survey of swine influenza literature [3,14,24,26]
ω	Average rate at which recovered animals become susceptible (for modeling reinfection)		
**VACCINATION PARAMETERS**			
	Transmission rate for pigs vaccinated with heterologous vaccine	0.0275 day^{-1}	Experimental data from Romagosa et al. [14] (mean of transmission values from heterologously vaccinated treatment group)
	Transmission rate for pigs vaccinated with homologous vaccine	0 day^{-1}	Experimental data from Romagosa et al. [14] (no transmission occurred to pigs in homologously vaccinated treatment group)
**PRODUCTION PARAMETERS**			
μ^{w}	Natural death rate of pigs	0.00028 day^{-1}	Corresponds to a death rate of 5% (from expert knowledge of swine farm; MT, personal comm.)

doi:10.1371/journal.pone.0106177.t003

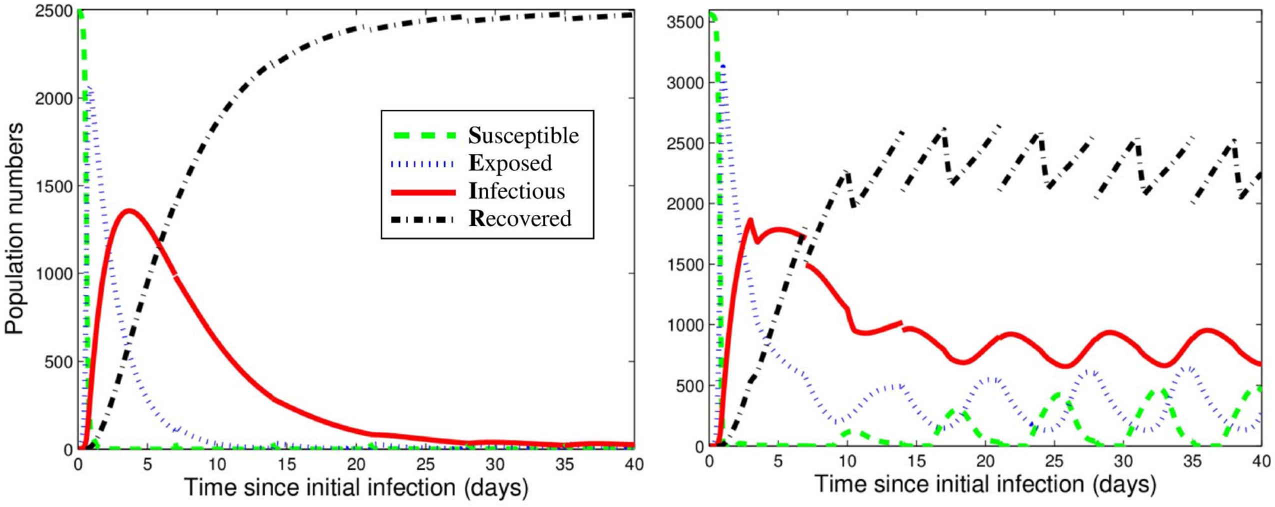

(a) Sows and gilts

(b) Piglets

Figure 2. Influenza dynamics as predicted by the breeding farm model, for (a) sows and gilts and (b) piglets, in a naïve (non-vaccinated) population. At time 0, one infectious gilt enters the breeding farm. Note that in panel (b) the piglets include both those with no maternal immunity and those with a reduced susceptibility due to maternal immunity. The discontinuities in the curves in these figures (and in subsequent figures) are caused by the weekly movement of swine through the farm or the removal of weaned piglets from the farm (as described in the Methods). The equilibrium dynamics are those after the initial peak in the number of infectious animals; these continue beyond the 40 days shown here.

doi:10.1371/journal.pone.0106177.g002

other conclusions made are not sensitive to variation in transmission.

Influenza Dynamics in the Wean-To-Finish Farm

Our model predicts that influenza infection spreads rapidly throughout the entire population of the wean-to-finish farm following the introduction of an infectious pig, and this is followed by disease extinction (Figure 5). For the results presented in Figure 5, we have assumed all pigs have maternal immunity. In this instance, the infection lasts for approximately 41 days in the population before disease extinction. In other scenarios, when the population has less maternal immunity (including the case when

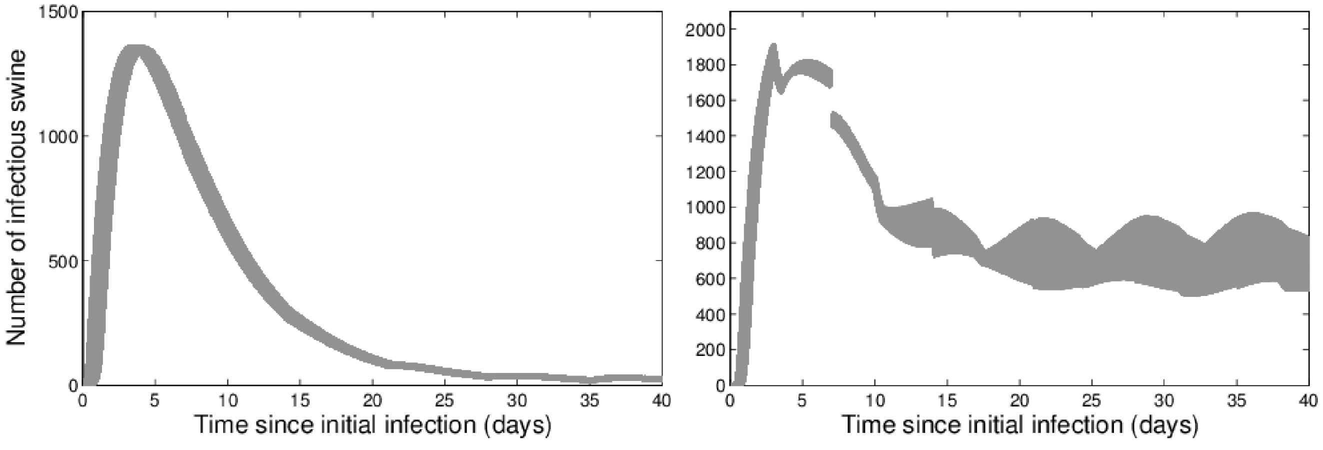

(a) Sows and gilts

(b) Piglets

Figure 3. Influenza infection dynamics as predicted by the breeding farm model with variability in transmission rates, for (a) sows and gilts and (b) piglets. The population of swine is naïve (non-vaccinated). At time 0, one infectious gilt enters the farm. These panels show the results of 15,000 runs, where for each run, all transmission rates are taken from random sampling from a uniform distribution spanning their 95% confidence intervals (Table 2).

doi:10.1371/journal.pone.0106177.g003

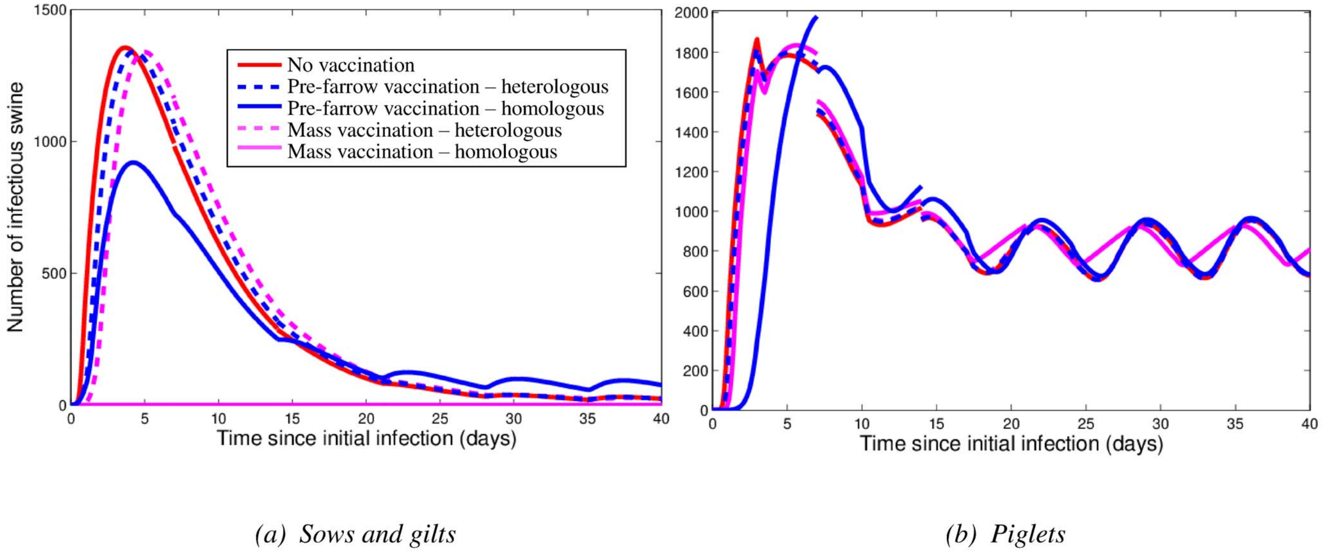

Figure 4. Summary of the effects of vaccination strategies on the number of infectious animals in the breeding farm, for (a) sows and gilts and (b) piglets. In (a), note that the 'Mass vaccination – homologous' curve lies along the x axis (as infection is eliminated). In (b), the 'Mass vaccination – heterologous' curve is mainly obscured by the 'Pre-farrow vaccination – heterologous' curve, which is very similar. For these results, vaccination occurs prior to the introduction of influenza.
doi:10.1371/journal.pone.0106177.g004

no pigs have maternal immunity), influenza spreads at a quicker rate, but the same shaped curves are observed (results not shown).

Reinfection. When the possibility of reinfection is incorporated into our wean-to-finish farm model, the influenza dynamics can change dramatically (Figure 6). For the first reinfection scenario, we assumed that animals that are infected early (specifically, in the two weeks after the introduction of influenza virus) are able to reenter the susceptible pool once recovered. Modeling this yields the results shown in Figure 6a, with a 'double peak' in the number of infectious animals. The second scenario

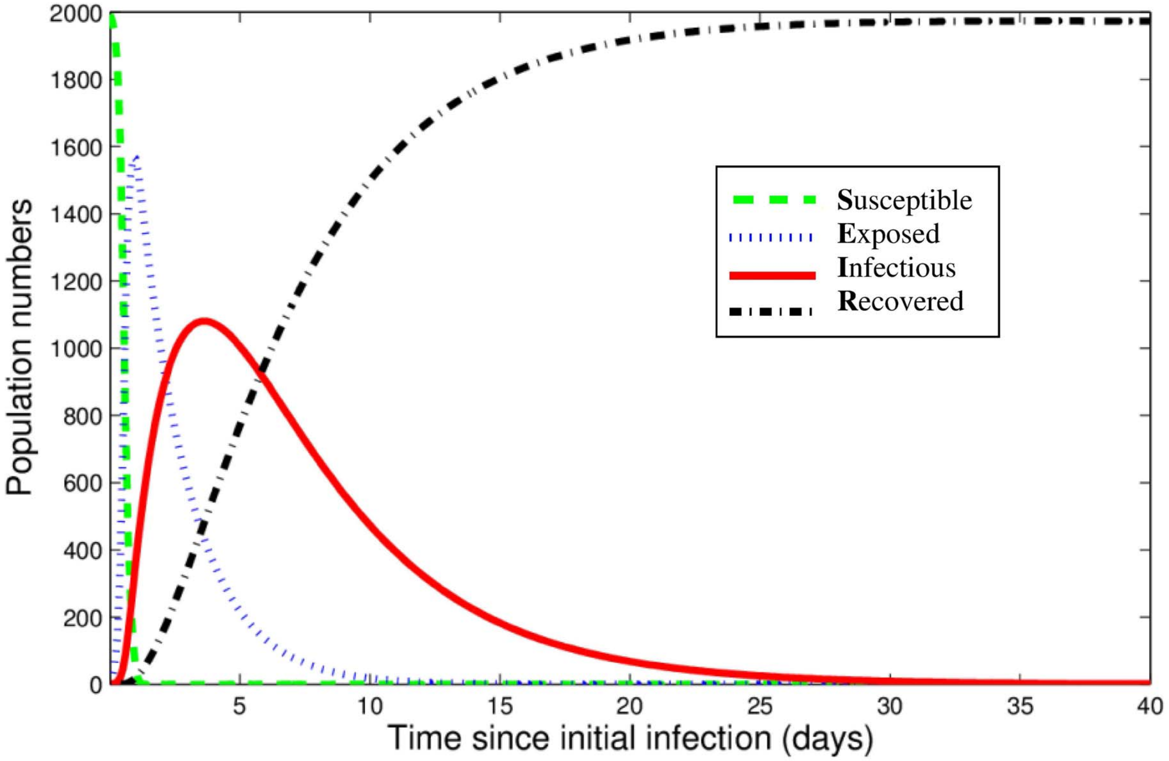

Figure 5. Results from the wean-to-finish farm model, when all pigs have maternal immunity (from immune mothers). At time 0, the farm becomes fully populated and one infectious pig enters.
doi:10.1371/journal.pone.0106177.g005

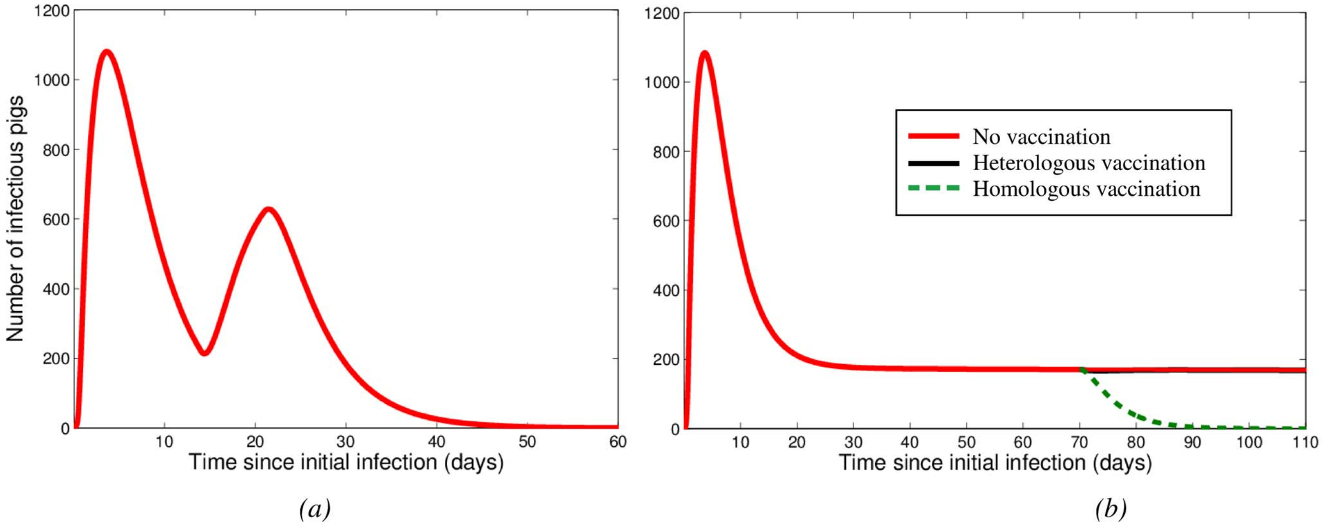

Figure 6. Model results for the wean-to-finish farm under two different reinfection assumptions. In (a), we show the number of infectious pigs in a population without vaccination and assume that pigs that are infected early can reenter the susceptible pool once recovered. In (b), we assume that recovered individuals can become susceptible again, due to either a change in the influenza virus, or through the loss of immunity. The average rate at which recovered animals move into the susceptible pool (ω) is $1/50$ in this example. Panel (b) also shows the number of infectious pigs when the population is vaccinated at $t=70$ (after maternal immunity has been lost). The heterologous vaccination at 70 days produces little effect on the number of infectious pigs.
doi:10.1371/journal.pone.0106177.g006

involved recovered animals becoming susceptible to infection at an average rate ω . As ω increases, the cumulative number of infectious animals increases. The long-term maintenance of influenza infection in the wean-to-finish population is possible with a sufficiently high value. Figure 6b without vaccination (red line) shows an example of the infection dynamics that could result in such a case; after the initial infection peak, there is a persistent level of infectious pigs.

Note that to fully understand these possible long-term dynamical behaviors, evolutionary information is required in order to predict and quantify the rate at which influenza virus changes. Further experimental verification is needed in order to determine the plausibility of the speculative dynamics illustrated in Figure 6.

Testing vaccination strategies. The results of vaccinating the entire population, after the loss of maternal immunity, are shown in Figure 6b. The homologous vaccine eliminates infection, whereas the heterologous vaccine has little effect on the number of infectious pigs. In Figures 5 and 6a, the infection has died out before pigs have lost their maternal immunity.

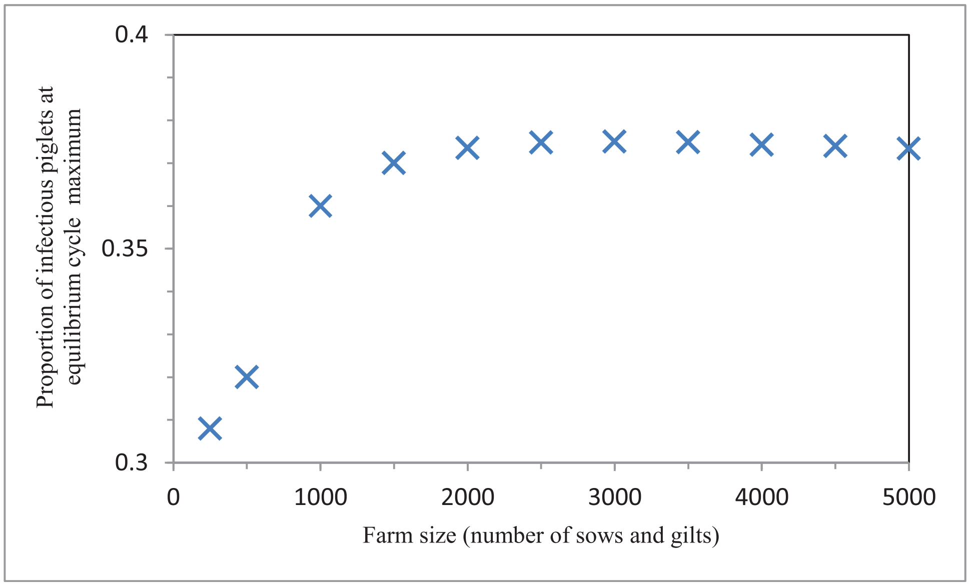

Figure 7. Breeding farm size effects on the proportion of infectious piglets at the maximum of the cycles at equilibrium. Farm size is defined as the number of sows and gilts on the farm.
doi:10.1371/journal.pone.0106177.g007

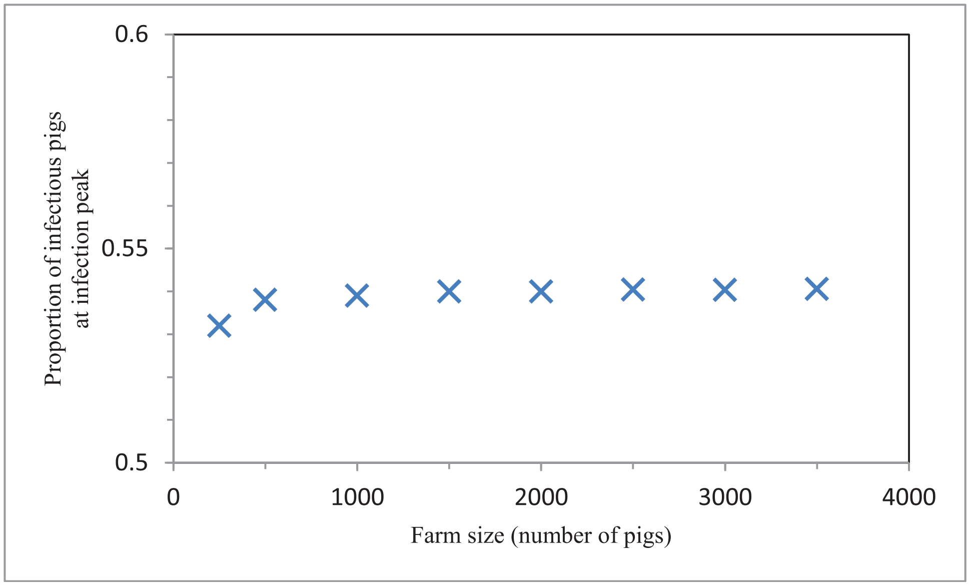

Figure 8. Wean-to-finish farm size effects on the proportion of infectious pigs at the infection peak. This proportion is a saturating function of farm size, defined as the number of pigs on the farm.
doi:10.1371/journal.pone.0106177.g008

Effects Of Farm Size on Influenza Dynamics

The maximum proportion of infectious piglets at equilibrium on the breeding farm varied from 0.31 to 0.38 across the range of farm sizes investigated (Figure 7). In addition, influenza spread at a quicker rate as the farm size increased. The proportion of infectious pigs at the infection peak on the wean-to-finish farm remained relatively constant as farm size changed, varying only from 0.53 to 0.54 (Figure 8).

Discussion

In this study, we developed a modeling framework to represent influenza transmission in breeding and wean-to-finish swine farms, informed by experimental data. This model provides the first theoretical description of influenza dynamics in swine at the population level. A key finding is that an influenza outbreak results in the maintenance through time of a high level of infectious piglets in breeding farms. The virus can be maintained in the piglet population because even when protected with maternal antibodies from immune sows, piglets are still susceptible to infection (albeit a reduced susceptibility), and new piglets are consistently born at regular intervals, providing a regular supply of susceptible piglets. The infection also persists in gilts, although at a lower level. Thus our model predicts that the introduction of influenza virus into a breeding farm results in endemicity. In the wean-to-finish facility, because there are no new susceptible animals entering the population once the farm is populated to sustain the infection through time, and as general theory predicts, influenza infection may persist in the closed population only if recovered individuals become susceptible to infection again.

Our theoretical predictions are similar to the limited field observations of influenza in swine farms. Inspired by the results of a recent empirical study [30], we tested whether piglets were the subpopulation of swine likely responsible for viral maintenance across a range of farm sizes and transmissibility rates. We found that our modeling results supported this hypothesis; we confirmed the finding that piglets had the highest infection levels on the breeding farm and that the infection levels were persistently high. In addition, Easterday and Hinshaw [37] reported that an influenza outbreak progresses through an entire herd within 2–3 weeks; our findings are consistent with this observation.

Our model results indicate that even the best current vaccination strategies are not sufficient to eliminate influenza throughout the entire breeding farm population. In particular, vaccination alone does not significantly reduce the level of infectious piglets, calling into question the profitability of current vaccination efforts, and suggesting that piglets should be targeted for further intervention in order to prevent the long-term persistence of influenza infection within a farm. However, if piglets themselves are vaccinated, maternal immunity inhibits the induction of active immunity and can reduce vaccine efficacy [36]. One potential solution is to vaccinate sows prior to insemination, so that they are protected themselves but do not pass down as much immunity to their offspring, followed with the subsequent vaccination of the piglets. Experimental quantification of influenza transmission for vaccinated unweaned piglets would be highly informative, and would allow us to test the effectiveness of this proposed strategy using our model.

Our model highlights the rapid spread of influenza within a population and the difficulty in limiting transmission once infection enters the farm. Perhaps swine producers should focus on eliminating sources of infection through increased biosecurity measures. This could involve multiple testing (and quarantining if necessary) of all gilts before they enter the breeding farm, and testing weaned pigs before entering the wean-to-finish facility. Because influenza can also be transmitted from humans to swine [38], also involved would be the prevention of influenza transmission from infected farm personnel, through measures such as vaccinating personnel, using masks, and avoiding work when showing flu symptoms. The high level of infectious piglets in the breeding farm indicates that the transport of weaned pigs to a wean-to-finish farm represents a risk of influenza transmission between these farms. Consistent with this prediction, transport of weaned pigs has been shown to disseminate influenza virus under field conditions [30,39].

We use transmission parameter values derived from experimental studies on swine influenza, which quantify the varying degrees of susceptibility among different groups of swine. However, there may also be differences in the infectiousness of different groups of swine, i.e. differences in the amount of shedding or the length of the infectious period, which affect the number of new animals becoming infected. For example, piglets with maternal immunity may differ in their infectiousness compared to those without maternal immunity [15]. Such heterogeneities in the levels of infectiousness have not been incorporated in the models presented here, and would be a valuable addition once variation in infectiousness has been experimentally demonstrated and quantified.

We have limited our focus to a single influenza strain in this study, and consequently limited our timescales. Over time, if influenza persists in the population, antigenic drift is possible [7]. The incorporation of mutation rates and evolutionary timescales, likely to be highly stochastic, would be a useful extension of our model framework. Then the longer-term dynamics of influenza may be explored. The inclusion of multiple influenza viral strains would also be an important addition, with different degrees of cross-immunity. Due to the stochastic nature of influenza infection, and also the differences depending on strain type, dynamics may vary from those predicted here. The intention of this study was to predict the dynamical trends of influenza infection; our interest resides in the qualitative patterns as opposed to exact quantitative predictions. In practice, the actual numbers of infectious pigs would be affected by a plethora of factors. For example, our assumption that the swine populations are naïve prior to the introduction of influenza has likely led to an overestimation of the numbers of infectious animals. Previous exposure to influenza is likely under field conditions given the ubiquitous nature of influenza infections in pigs.

Sensitivity analysis was performed to test the robustness of our findings to both uncertainties in transmission estimates and also variation in farm size. Our main conclusions were robust to changes in transmission rates within their 95% confidence intervals. Although decreasing the breeding farm size below 1500 sows and gilts caused a minor decrease in the maximum proportion of infectious piglets at equilibrium, there was still endemicity; even for the smallest of farm sizes suitable for U.S. commercial operations, there was a high proportion of infectious piglets that persisted through time. Changing the wean-to-finish farm size had very little effect on the proportion of infectious pigs at the infection peak.

The maintenance of influenza infection predicted by our model, especially in 18–27% of piglets on the breeding farm, confirms that pig populations represent a potential threat to public health. The longer a strain is maintained in a population, and the greater the number of infectious animals, the greater the probability of transmission to humans and the greater the chance of virus evolution and reassortment, thus enabling species jumps. Although swine farmers have access to an extensive set of intervention strategies that can reduce or eliminate other pathogens [40], successful interventions have yet to be devised for influenza. Our model predicts that the most common influenza vaccination strategies are ineffective in eliminating, or even reducing, influenza infection in a breeding herd, as the virus can still be found in piglets regardless of target population or vaccine type. Our study suggests that control strategies should focus on increasing biosecurity to prevent new introductions of influenza as well as mitigating infection in piglets. There is an untapped opportunity to use mathematical modeling combined with experimental studies to devise successful control strategies for influenza, and more broadly, to integrate public health concerns into animal production decisions.

Supporting Information

Figure S1 Summary of the effects of vaccination strategies on the number of infectious animals in the breeding farm, with vaccination occurring when influenza is endemic and circulating in the population (i.e. after an outbreak). In (a), note that the 'Mass vaccination – homologous' curve lies along the x axis (as infection is eliminated). In addition, both pre-farrow curves are obscured by the 'No vaccination' curve, which is very similar in both cases. In (b), all curves are very similar to the 'No vaccination' curve; vaccination does not measurably affect the number of infectious piglets.
(EPS)

Acknowledgments

This material is based upon work supported by the University of Minnesota's Institute on the Environment and the Cooperative State Research Service, U.S. Department of Agriculture, under Project No. MINV 62-042. Any opinions, findings, conclusions or recommendations expressed in this publication are those of the authors and do not necessarily reflect the view of the U.S. Department of Agriculture. The authors would like to thank Dr Samuel Baidoo and Dr Matt Allerson for advice.

Author Contributions

Conceived and designed the experiments: MC MT JR. Performed the experiments: JR. Analyzed the data: JR MC. Contributed reagents/materials/analysis tools: MC MT JR. Contributed to the writing of the manuscript: JR MC MT.

References

1. Webster RG. (2002) The importance of animal influenza for human disease. Vaccine 20: S16–S20.
2. Ma W, Kahn RE, Richt JA. (2009) The pig as a mixing vessel for influenza viruses: Human and veterinary implications. J Mol Genet Med 3: 158–166.
3. Brown IH. (2000) The epidemiology and evolution of influenza viruses in pigs. Vet Microbiol 74: 29–46.
4. Vincent A, Awada L, Brown I, Chen H, Claes F, et al. (2014) Review of influenza A virus in swine worldwide: A call for increased surveillance and research. Zoonoses and Public Health 61: 4–17.
5. Torremorell M, Allerson M, Corzo C, Diaz A, Gramer M. (2012) Transmission of influenza A virus in pigs. Transboundary and Emerging Diseases 59: 68–84.
6. Van Reeth K, Brown IH, Olsen CW. (2012) Influenza virus. In: Zimmerman J, Karriker L, Ramirez A, Schwartz K, Stevenson G, editors. Diseases of Swine. Hobokin, NJ: Wiley-Blackwell. pp. 557–571.
7. Olsen CW. (2002) The emergence of novel swine influenza viruses in North America. Virus Res 85: 199–210.
8. Shope RE. (1931) Swine influenza: iii. Filtration experiments and etiology. J Exp Med 54: 373–385.
9. Tellier R. (2006) Review of aerosol transmission of influenza A virus. Emerg Infect Dis 12: 1657–1662.
10. Allerson MW, Cardona CJ, Torremorell M. (2013) Indirect transmission of influenza A virus between pig populations under two different biosecurity settings. PLoS One 8: e67293.
11. Vincent AL, Ma W, Lager KM, Janke BH, Richt JA. (2008) Swine influenza viruses: a North American perspective. Adv Virus Res 72: 127–154.
12. USDA. (2007) Swine 2006, part II: Reference of swine health and health management practices in the United States, 2006.
13. Allerson M, Deen J, Detmer SE, Gramer MR, Joo HS, et al. (2013) The impact of maternally derived immunity on influenza A virus transmission in neonatal pig populations. Vaccine 31: 500–505.
14. Romagosa A, Allerson M, Gramer M, Joo HS, Deen J, et al. (2011) Vaccination of influenza a virus decreases transmission rates in pigs. Vet Res 42: 120–9716–42–120.
15. Loeffen WL, Heinen PP, Bianchi AT, Hunneman WA, Verheijden JH. (2003) Effect of maternally derived antibodies on the clinical signs and immune

response in pigs after primary and secondary infection with an influenza H1N1 virus. Vet Immunol Immunopathol 92: 23–35.
16. Markowska-Daniel I, Pomorska-Mol M, Pejsak Z. (2011) The influence of age and maternal antibodies on the postvaccinal response against swine influenza viruses in pigs. Vet Immunol Immunopathol 142: 81–86.
17. Lloyd-Smith JO, George D, Pepin KM, Pitzer VE, Pulliam JR, et al. (2009) Epidemic dynamics at the human-animal interface. Science 326: 1362–1367.
18. Pulliam JRC, Epstein JH, Dushoff J, Rahman SA, Bunning M, et al. (2012) Agricultural intensification, priming for persistence and the emergence of Nipah virus: A lethal bat-borne zoonosis. Journal of the Royal Society Interface 9: 89–101.
19. Grenfell BT, Smith G. (1990) Mathematical model for the impact of a pseudorabies epizootic on the productivity of a farrow-to-finish operation. Am J Vet Res 51: 156–164.
20. Smith G, Grenfell BT. (1990) Population biology of pseudorabies in swine. Am J Vet Res 51: 148–155.
21. Evans CM, Medley GF, Creasey SJ, Green LE. (2010) A stochastic mathematical model of the within-herd transmission dynamics of porcine reproductive and respiratory syndrome virus (PRRSV): Fade-out and persistence. Prev Vet Med 93: 248–257.
22. Ivanek R, Snary EL, Cook AJ, Grohn YT. (2004) A mathematical model for the transmission of Salmonella Typhimurium within a grower-finisher pig herd in Great Britain. J Food Prot 67: 2403–2409.
23. Lurette A, Belloc C, Touzeau S, Hoch T, Ezanno P, et al. (2008) Modelling Salmonella spread within a farrow-to-finish pig herd. Vet Res 39: 49.
24. Dorjee S, Poljak Z, Revie CW, Bridgland J, McNab B, et al. (2013) A review of simulation modelling approaches used for the spread of zoonotic influenza viruses in animal and human populations. Zoonoses Public Health 60: 383–411.
25. Real L, Biek R. (2007) Infectious disease modeling and the dynamics of transmission. In: Childs JE, Mackenzie JS, Richt JA, editors. Wildlife and emerging zoonotic diseases: the biology, circumstances and consequences of cross-species transmission. New York: Springer. pp. 33–49.
26. Saenz RA, Hethcote HW, Gray GC. (2006) Confined animal feeding operations as amplifiers of influenza. Vector Borne Zoonotic Dis 6: 338–346.
27. Flahault A, Deguen S, Valleron AJ. (1994) A mathematical model for the European spread of influenza. Eur J Epidemiol 10: 471–474.
28. Casagrandi R, Bolzoni L, Levin SA, Andreasen V. (2006) The SIRC model and influenza A. Math Biosci 200: 152–169.
29. Coburn BJ, Wagner BG, Blower S. (2009) Modeling influenza epidemics and pandemics: Insights into the future of swine flu (H1N1). BMC Med 7: 30-7015-7-30.
30. Allerson MW, Davies PR, Gramer MR, Torremorell M. (2013) Infection dynamics of pandemic 2009 H1N1 influenza virus in a two-site swine herd. Transbound Emerg Dis. doi: 10.1111/tbed.12053.
31. Hanski I, Gilpin M. (1991) Metapopulation dynamics: Brief history and conceptual domain. Biol J Linn Soc 42: 3–16.
32. Hanski IA, Gaggiotti OE, editors. (2004) Ecology, genetics, and evolution of metapopulations. Amsterdam: Elsevier Academic Press.
33. Anderson RM, May RM. (1979) Population biology of infectious diseases: Part I. Nature 280: 361–367.
34. Acha PN, Szyfres B. (2003) Influenza. In: Zoonoses and communicable diseases common to man and animals. Volume 2. Chlamydiosis, rickettsioses and viroses. Washington DC: Pan American Health Organization. pp. 155–172.
35. Easterday BC. (1971) Influenza virus infection of the suckling pig. Acta Veterinaria Brno 2: 33–42.
36. Kitikoon P, Nilubol D, Erickson BJ, Janke BH, Hoover TC, et al. (2006) The immune response and maternal antibody interference to a heterologous H1N1 swine influenza virus infection following vaccination. Vet Immunol Immunopathol 112: 117–128.
37. Easterday BC, Hinshaw VS. (1992) Swine influenza. In: Leman AD, Straw BE, Mengeling WL, D'Allaire SD, Taylor DJJ, editors. Diseases of Swine. Ames, IA: Iowa State Press. pp. 349–357.
38. Nelson MI, Gramer MR, Vincent AL, Holmes EC. (2012) Global transmission of influenza viruses from humans to swine. J Gen Virol 93: 2195–2203.
39. Nelson M, Lemey P, Tan Y, Vincent A, Lam T, et al. (2011) Spatial dynamics of human-origin H1 influenza A virus in North American swine. PLoS Pathogens 7: e1002077.
40. Corzo CA, Mondaca E, Wayne S, Torremorell M, Dee S, et al. (2010) Control and elimination of porcine reproductive and respiratory syndrome virus. Virus Res 154: 185–192.
